# Joint-specific regulation of homeobox D10 expression in rheumatoid arthritis fibroblast-like synoviocytes

**DOI:** 10.1371/journal.pone.0304530

**Published:** 2024-06-03

**Authors:** Hyeonjeong Lee, Camilla R. L. Machado, Deepa Hammaker, Eunice Choi, Edward B. Prideaux, Wei Wang, David L. Boyle, Gary S. Firestein

**Affiliations:** 1 Division of Rheumatology, Allergy and Immunology, School of Medicine, University of California San Diego, La Jolla, California, United States of America; 2 Department of Chemistry and Biochemistry, University of California San Diego, La Jolla, California, United States of America; University of Rijeka Faculty of Medicine: Sveuciliste u Rijeci Medicinski fakultet, CROATIA

## Abstract

Rheumatoid arthritis (RA) is a systemic immune-mediated disease characterized by joint inflammation and destruction. The disease typically affects small joints in the hands and feet, later progressing to involve larger joints such as the knees, shoulders, and hips. While the reasons for these joint-specific differences are unclear, distinct epigenetic patterns associated with joint location have been reported. In this study, we evaluated the unique epigenetic landscapes of fibroblast-like synoviocytes (FLS) from hip and knee synovium in RA patients, focusing on the expression and regulation of Homeobox (HOX) transcription factors. These highly conserved genes play a critical role in embryonic development and are known to maintain distinct expression patterns in various adult tissues. We found that several HOX genes, especially HOXD10, were differentially expressed in knee FLS compared with hip FLS. Epigenetic differences in chromatin accessibility and histone marks were observed in HOXD10 promoter between knee and hip FLS. Histone modification, particularly histone acetylation, was identified as an important regulator of HOXD10 expression. To understand the mechanism of differential HOXD10 expression, we inhibited histone deacetylases (HDACs) with small molecules and siRNA. We found that HDAC1 blockade or deficiency normalized the joint-specific HOXD10 expression patterns. These observations suggest that epigenetic differences, specifically histone acetylation related to increased HDAC1 expression, play a crucial role in joint-specific HOXD10 expression. Understanding these mechanisms could provide insights into the regional aspects of RA and potentially lead to therapeutic strategies targeting specific patterns of joint involvement during the course of disease.

## Introduction

Rheumatoid arthritis (RA) is a systemic immune-mediated disease characterized by joint inflammation and destruction [1, 2]. The distribution of RA is symmetrical, often beginning in the small joints of hands and feet and then involving the larger joints like knees, shoulders and hip [3]. While the reason for joint-specific distribution is not well known, distinct epigenetics patterns based on joint location have been reported [4, 5]. For example, we and others previously identified the DNA methylation and transcriptome signatures in fibroblast-like synoviocytes (FLS) derived from hip vs. knee joints in patients with RA [4–6]. RA FLS are mesenchymal resident cell in the synovial intimal lining and exhibit an aggressive phenotype [7, 8]. This activated phenotype includes enhanced proliferation, resistance to apoptosis, and production cytokines and proteases that promote joint damage. In some cases, this aberrant synovial fibroblast behavior results from a response to the local cell environment [9, 10]. However, some behaviors are likely caused by differential epigenetic marks in genes and pathways that contribute to this phenotype [11, 12].

Our studies to understand the joint-specific phenotype of FLS led us to homeobox (HOX) transcription factors. HOX genes are highly conserved and play a pivotal role in morphogenesis and limb formation during embryonic development [13, 14]. In many cases, these expression patterns are maintained in adult cells and tissues where they can contribute the tissue homeostasis [5, 15–18]. Review of transcriptome and epigenetic data in RA and osteoarthritis revealed remarkable differences in multiple HOX genes between cells derived from hips, knees and other locations in both diseases [4, 5].

In this study, we focused on RA and evaluated the mechanisms of HOX genes regulation and differential expression in hip compared with knee FLS. We focused on HOXD10 because knee FLS expression is typically more than 100-fold higher than in hip FLS. Our data suggest that these differences are due to epigenetic modifications in the HOXD10 promoter, particularly histone modifications through acetylation. These results provide an explanation for differential HOX gene expression and also show that the differences can be regulated by remodeling the FLS epigenome.

## Materials and methods

### Synovial tissue and FLS from rheumatoid arthritis patients

Synovial tissue was obtained from RA patients during total joint replacement or synovectomy. RA patients were diagnosed following the American College of Rheumatology 2010 criteria [19]. Clinical information beyond diagnosis, age and joint of origin was unavailable on these de-identified samples. The Human Research Protection approved the protocol (#14–0175), and informed consent was obtained from all patients. FLS were established from synovial tissues as previously described [11] and used from passage 5 through 8. Cells were cultured in Gibco Dulbecco’s Modified Eagle Medium (DMEM) supplemented with penicillin/streptomycin, L‐glutamine, and gentamicin, (complete DMEM), and 10% heat‐inactivated Fetal Bovine Serum (FBS) at humidified 5% CO2 atmosphere.

### Quantitative real-time PCR

Total RNA from synovial tissues and FLS was performed using RNeasy mini kit (Qiagen) following the manufacturer’s instruction. The complementary DNA (cDNA) was synthesized from 250-500ng of RNA using TaqMan reverse transcription reagents (Thermo Fisher Scientific), and qPCR was carried out using primer and probe set (Thermo Fisher Scientific) and StepOne™ Real‐Time PCR System (Thermo Fisher Scientific). GAPDH was used for control, and relative gene expression was calculated using the comparative 2^−ΔΔCt^ method [20].

### Immunohistochemistry

Cryosections (5 μm) of synovial tissue were fixed in acetone for 10 min and prepared as previously described [21]. The sections were blocked with diluted normal horse serum (Vector laboratory) for 20 minutes, followed by overnight incubation with primary mouse anti-HOXD10 antibody (1:450; Invitrogen; MA5-26485) at 4°C. After washing, diluted biotinylated secondary antibody (Vector laboratory) was added for 30 minutes, followed peroxidase substrate solution (Vector laboratory). Sections were counter-stained with Mayer’s hematoxylin (Fisher), washed in water, and mounted in aqueous mounting medium (Vector laboratory). Quantification analysis for DAP-positive were calculated by Image J program [22].

### Plasmid construction and luciferase reporter assay

The HOXD10 promoter fragment (1-kbp) was amplified from genomic DNA of FLS with designed primers by PCR. Primers were 5’-CGATGCTAGCGCCCAGTCAGTTTTCTAAAAC-3’ (forward) and 5’-CGATAGATCTTTGTGTCCTTTGTAGGCTGAC-3’ (reverse). The fragment was subcloned into the pGL4.23 vector (Promega). The construct was confirmed by sequencing (Eton Bioscience). 1 μg of HOXD10 promoter plasmid was co-transfected with Renilla plasmid into FLS. After 24 h, FLS were treated with 50 ng/ml TNF for 2 h, and then lysed. The luciferase activity was determined using Dual-Luciferase Reporter Assay System (Promega) according to the manufacturer’s instruction. The relative luciferase activity was normalized to Renilla activity [23].

### mRNA stability assay

To measure the decay rate of HOXD10 mRNA in hip and knee FLS, cells were treated with 10 μg/ml actinomycin D for a maximum of 12 h, harvested at 1 h, 3 h, 6 h, and 12 h for RNA isolation, and analyzed by qPCR and normalized with GAPDH gene expression.

### Gene silencing

5 x 10^5^ FLS were transfected with 1 μg of HOTAIR, HDAC1, HDAC3 (small interfering Smart Pool On-Target RNA, Horizon Discovery) or non-targeting control pool siRNA (Horizon Discovery) using the normal human dermal fibroblast Nucleofactor kit (Lonza), according to the manufacturer’s instruction [24]. Silencing efficiencies of HOTAIR, HDAC1 and HDAC3 were 71%, 82% and 88%, respectively.

### ATAC-seq and ChIP-seq analysis

The procedure for generation and processing ATAC‐seq (performed by the Center for Epigenetics, UCSD) and ChIP-seq data, were performed as previously described [11]. Four hip FLS and five knee FLS datasets from those data were available for subsequent analysis. To determine the differentially accessible regions (ATAC-seq) and differentially modified epigenetic region (DMER, ChIP-seq) between hip and knee FLS, DiffBind package in R (FDR < 0.05) was used. The data used in this study are available in Gene Expression Omnibus with the primary accession code GSE112658.

### Chromatin immunoprecipitation (ChIP)-qPCR

ChIP assays were conducted using a Zymo-Spin ChIP kit (Zymo Research) according to the manufacturer’s instruction. Briefly, 5 x 10^6^ FLS were cross-linked with 1% ChIP-grade formaldehyde (Sigma), lysed, and then sonicated to shear the chromatin to fragment sizes of 200–700 bp. The sheared chromatin was immunoprecipitated with 2 μg H3K27ac antibody (Diagenode; C15410196) or Rabbit IgG (Diagenode; C15410206) at 4°C, eluted, and reverse cross-linked. Eluted DNA was analyzed by qPCR with specific primer set and Power SYBR green Mastermix (Thermo Fisher Scientific). Primers were 5’-GAATCCGACTCACCTTCCC-3’ (forward) and 5’-ACACAACCAGGCAGAACG-3’ (reverse), and amplification size was 210 bp.

### Western blot

FLS were treated with HDAC inhibitors (10 μM MS-275 or 500 nM ITF2357) for 12 h and 24 h, and Western blot analysis was performed as previously described [24]. Protein concentration was measured using the Micro BCA™ Protein Assay Kit (Thermo Fisher Scientific), and 25 μg of protein was loaded onto SDS-PAGE gel. The proteins were transferred from the gel to the PVDF membrane. The membrane was blocked with 5% skim milk, followed by overnight incubation with H3K27ac antibody (Abcam; ab4729), or α-tubulin antibody (Cell Signaling Technology; #3873) at 4°C. Horseradish peroxidase (HRP)‐conjugated goat anti‐rabbit IgG or anti‐mouse (Cell Signaling Technology; #7074 and #7074) were used as secondary antibody. The signal was developed by Immun‐Star WesternC ECL substrate (BioRad), captured using a VersaDoc imaging system, and then analyzed using Image J. Protein expression level was normalized to α-tubulin.

### Statistical analysis

Statistics were carried out by two-way t test, Mann-Whitney test, or one-way analysis of variance (ANOVA) followed by Tukey’s test for multiple comparisons using GraphPad Prism 9. P values < 0.05 were considered significant.

## Results

### Differential HOX expression in hip and knee FLS

**Fig 1:**
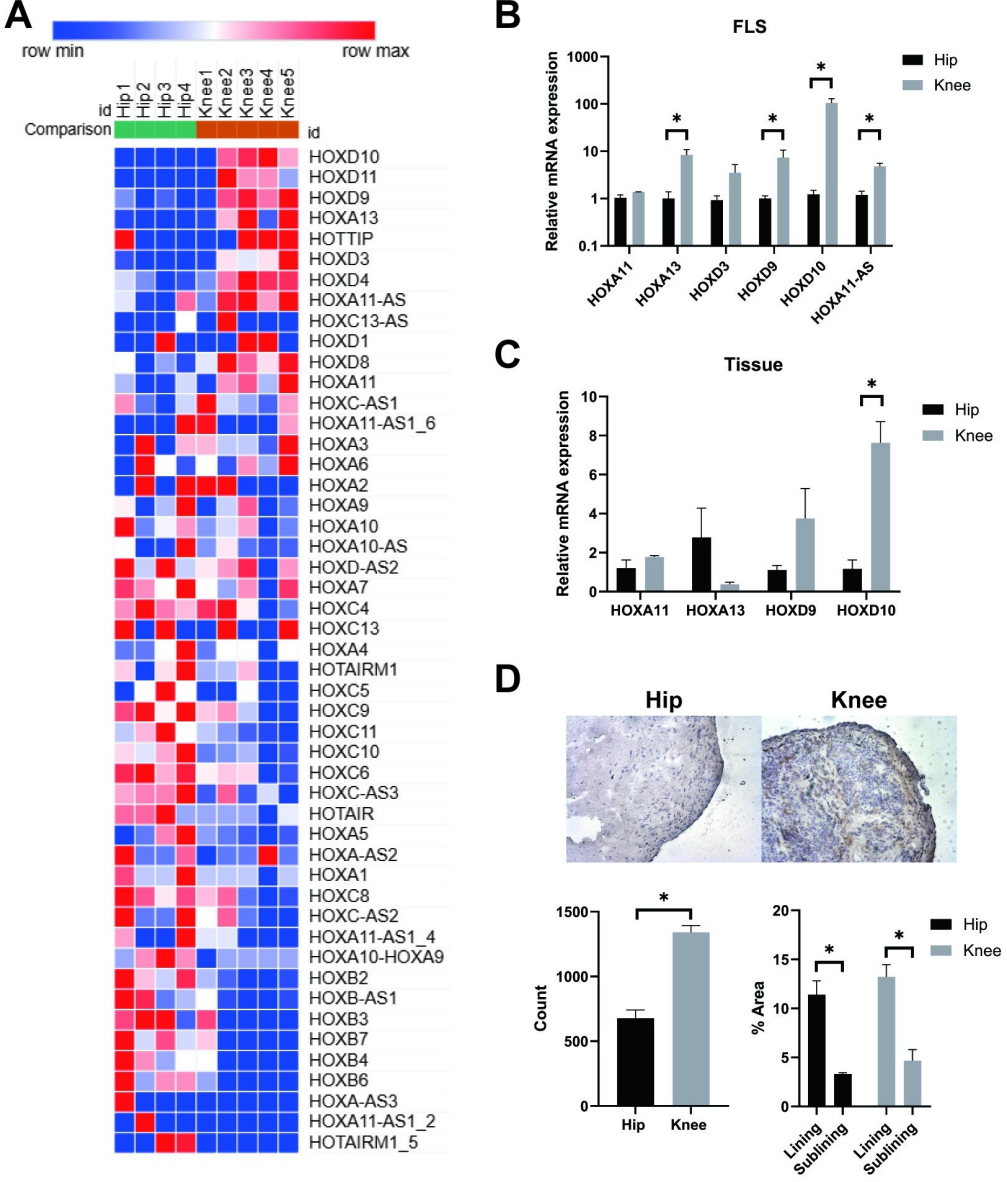
HOX expression in RA FLS and synovial tissue. (A) Heatmap of HOXs genes for RA hip (green) and knee (orange) FLS. Many HOX genes were differentially expressed between hip and knee FLS. For example, HOXA13, HOXD3, HOXD9, and especially HOXD10 were significantly higher in knee FLS than hip FLS. (B) Expression of various HOXs mRNA was measured by qRT-PCR in RA hip (n = 4) and knee (n = 4) FLS. Similar to heat map visualization, HOXA13, HOXDD9, and HOXA11-AS expression were significantly higher in knee FLS than hip FLS. HOXD10 showed markedly higher expression in knee FLS. Data were presented as mean± SEM and analyzed using the two-tailed t-test. *P<0.05. (C) Expression of various HOXs mRNA in RA hip (n = 4) and knee (n = 4) tissue. As with mRNA expression in RA FLS, knee synovium expressed HOXD10 significantly higher than hip synovium. Data were presented as mean± SEM and analyzed using the two-tailed t-test. *P<0.05. (D) Representative microscopic image (magnification, x200) of immunohistochemistry in hip and knee joint tissues for the expression of HOXD10. HOXD10 protein level in knee synovial tissue was significantly higher compared with hip’s and HOXD10 was mainly expressed in the intimal lining region. Data were presented as mean± SEM on bar graphs and analyzed using the two-tailed t-test. *P<0.05.

Previous studies suggested that HOX genes, most notably HOXD10, might be differentially expressed in hip and knee synovium [4]. To profile this finding more completely, we used our published FLS RNAseq database to compare the relative expression levels of HOX genes and rank them by the hip:knee ratio (See Fig 1A [21]). To confirm those observations, we evaluated a representative group of HOX genes that showed varying degree of differences between RA hip and knee-derived FLS by RT-qPCR. Fig 1B shows that HOXA 11 and HOXD3 mRNA expression was similar in FLS derived from either joint (n = 4). However, several HOX genes were more highly expressed in RA knee FLS compared with knee FLS, including HOXA11, HOXD9, HOXD10, HOXA11-AS. Of these, HOXD10 showed the greatest difference and was 85±20-fold higher in RA knee FLS compared to hip FLS (*P* = 0.009).

### Differential HOX expression in hip and knee synovial tissue

Because HOXD10 expression is markedly higher in knee FLS, we focused our subsequent experiments on understanding differential regulation. Initially, we evaluated RNA and protein levels in hip and knee synovium. Fig 1C shows HOXD10 gene expression in knee synovial tissue extracts were 8.6±0.93-fold higher than hip tissue (*P* = 0.005, n = 4). We then examined HOXD10 protein expression in the intact tissue by IHC and found that it is higher in knee than hip tissue (*P* = 0.0002, n = 4, left panel of Fig 1D). HOXD10 protein was found throughout the synovium but was more intense in the intimal lining where FLS reside (*P* = 0.0013 and 0.0023, respectively, n = 4, right panel of Fig 1D).

### Regulation of HOX genes in FLS

**Fig 2:**
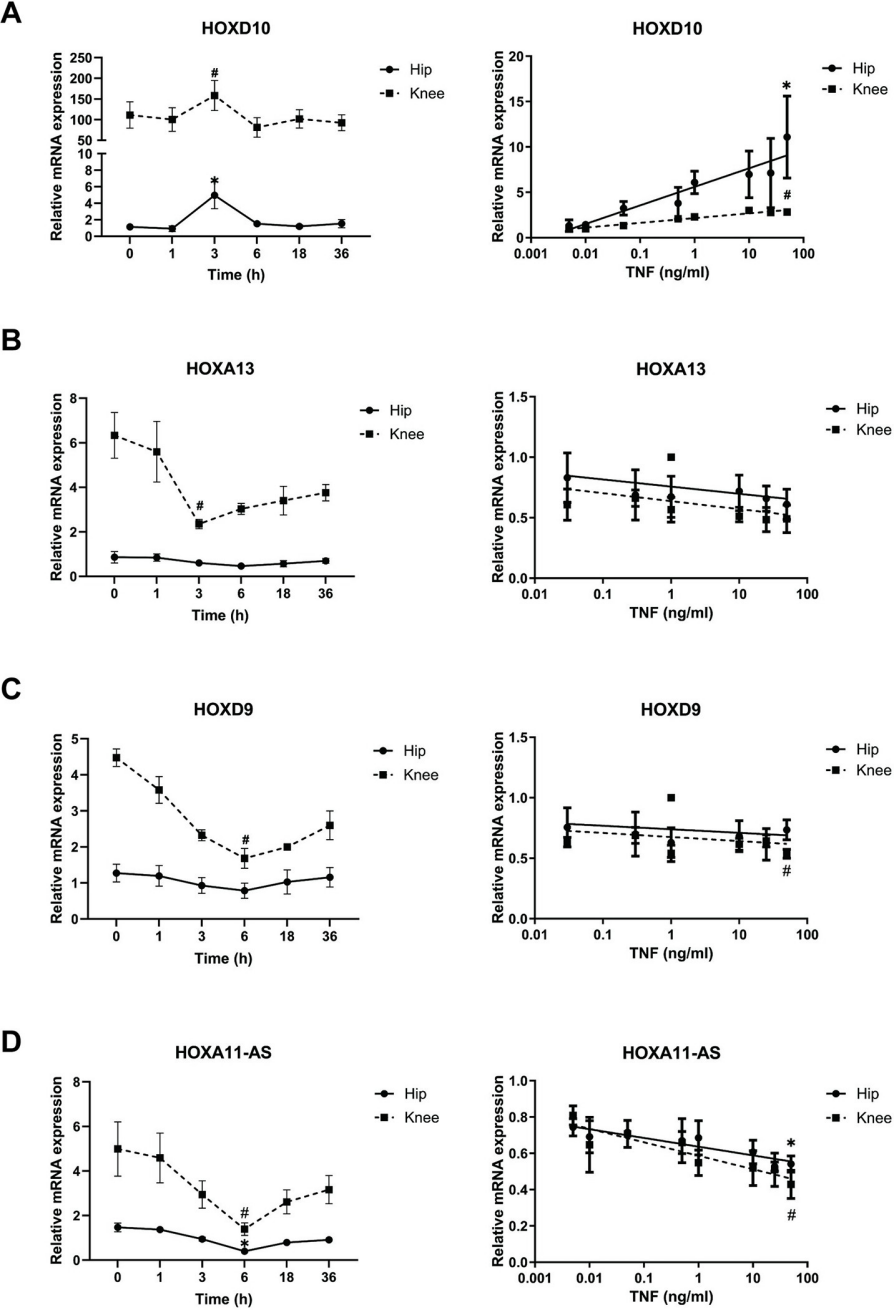
Regulation of HOXs in RA FLS. (A-D) HOX genes were measured by qRT-PCR at various times after 50 ng/ml TNF stimulation (left panel) and with various concentration of TNF (right panels) (n = 3). Right panel graphs show fold change value compared with non-treated RA FLS. After 3 h, HOXD10 expression was significantly higher at 50 ng/ml TNF. Data were presented as mean± SEM and analyzed using the two-tailed t-test. * and # *P*<0.05.

We then evaluated how HOX genes are regulated by TNF, which is a critical mediator of the inflammatory pathway in RA and could potentially explain differential expression. As shown in Fig 2A, TNF increased HOXD10 expression in both hip and knee FLS, with peak expression after 3 h (n = 3). In contrast, HOXA13, HOXD9, and HOXA11-AS expression was modestly decreased by TNF with lowest levels at 3 h, 6 h, and 6 h, respectively (Fig 2B and 2C). The ratios of HOXD10 expression were similar between hip and knee FLS after TNF stimulation. The knee-hip ratio was modestly decreased for HOXA13, HOXD9, and HOXA11-AS, although there was still greater expression in knee FLS. Of interest, MMP3 and IL-8 gene expression in hip and knee significantly correlated with HOXD10 expression (S2 Fig).

### Regulation of HOXD10 transcript in FLS

**Fig 3:**
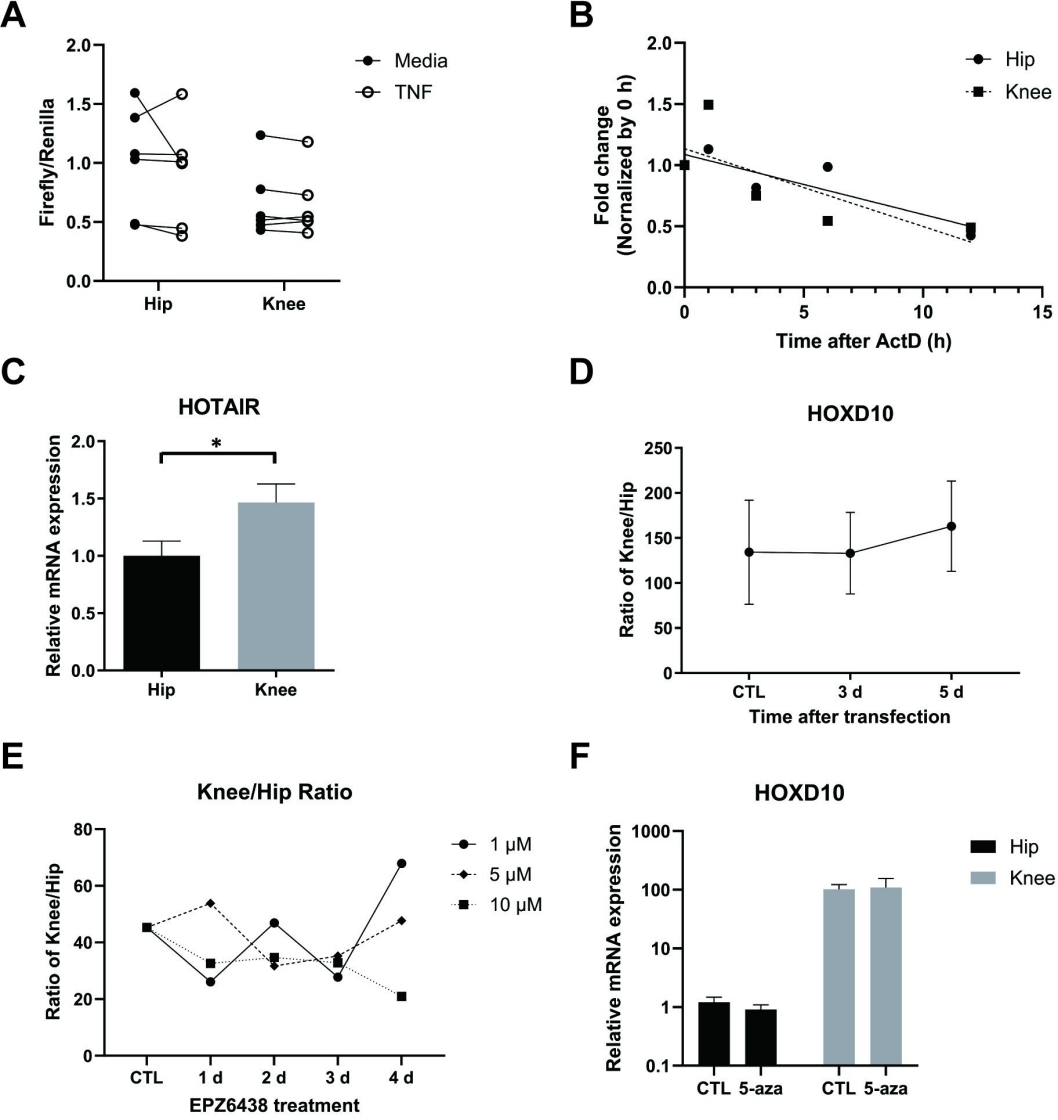
Regulation of HOXs transcript in RA FLS. (A) The NF-kB reporter construct and renilla vector were co-transfected and RA FLS and then stimulated with 50 ng/ml TNF for 3 h (n = 6). Luciferase activity was normalized by ranilla activity. Knee and hip FLS showed comparable HOXD10 promoter activity in the presence or absence of TNF. Data were analyzed using the two-tailed t-test. *P<0.05. (B) RA FLS were incubated with 10 μg/ml actinomycin for various times and HOXD10 mRNA was measured by qRT-PCR (n = 5). Data are presented as fold change compared with 0 h and analyzed using simple linear regression. (C) Expression of HOTAIR was measured by qRT-PCR in hip (n = 6) and knee FLS (n = 6). HOTAIR expression was significantly higher in knee FLS than hip FLS. Data were presented as mean± SEM, and analyzed using the two-tailed t-test. *P<0.05. (D) After siRNA targeting HOTAIR transfection, HOXD10 mRNA was measured by qRT-PCR (n = 3). After transfection, the ratio of HOXD10 expression in knee FLS and hip FLS was modestly increased. (E) HOXD10 mRNA level was measured at various time points with 1–10 μM EPZ6438 (n = 6). The ratio of HOXD10 expression in knee FLS and hip FLS did not show a significant difference after stimulation. (D, E) Data was analyzed by dividing HOXD10 expression of knee FLS by one of hip FLS and analyzed using the two-tailed t-test. (F) After DNA methyltransferase (DNMT) inhibitor treatment for 2 weeks, HOXD10 expression was measured by qRT-PCR (n = 6). Knee and hip FLS displayed comparable HOXD10 transcript in the presence or absence of DNMT inhibitor (n = 7). Data was presented as mean± SEM, and analyzed using the two-tailed t-test.

The mechanism of increased HOXD10 gene expression in knee compared to hip FLS was then evaluated. Initial experiments used an HOXD10 promoter-reporter construct to determine whether increased knee FLS transcription is responsible. Fig 3A shows that knee and hip FLS displayed comparable HOXD10 promoter activity in the presence or absence of TNF (n = 6). We then considered the potential role of post-transcriptional regulation such as mRNA degradation. However, HOXD10 mRNA half-life was similar for knee (9.2 h) and hip (11.6 h) FLS (*P* > 0.10, n = 5, Fig 3B). Previous studies have reported that the lncRNA HOTAIR regulates the expression of multiple HOX genes, including HOXD10. Knee FLS showed higher expression of HOTAIR than hip FLS (*P* < 0.0471, n = 6, Fig 3C). Furthermore, HOTAIR knockdown did not eliminate the differences between hip and knee FLS and, in fact, the reduction HOTAIR led to a trend towards a modest increase the ratio of HOXD10 expression (n = 3, Fig 3D). These data indicate that differential expression of HOTAIR is not responsible for the differences on HOXD10 expression between these two joint locations.

### Regulation of HOXD10 transcript via histone and DNA methylation in RA FLS

Because the promoter-reporter construct, mRNA stability and HOTAIR knockdown studies did not identify the mechanism of differential HOXD10 expression, we considered whether epigenetic marks contributed. We first investigated whether histone methylation contributes to differential expression of HOXD10 based on joint location. We treated FLS with EPZ6438, a selective EZH2 inhibitor, to suppress H3K27 methylation. Fig 3E shows that the inhibitor had no effect on joint-specific the ratio of knee and hip FLS HOXD10 expression (n = 6, Fig 3E). We then cultured FLS for 2 weeks in the presence of a DNA methyltransferase inhibitor 5-aza-2’-deoxycytidine to determine if a global decrease in DNA methylation was responsible. The inhibitor did not affect HOXD10 mRNA expression in either joint (n = 7, Fig 3F).

### Differential epigenetic profiles in hip and knee FLS

Chromatin accessibility and histone peaks in HOXD10 promoter regions were then examined using our ATAC-seq and ChIP-seq database. Of interest, RA knee FLS had greater accessibility in HOXD10 promoter region compared with hip FLS (P = 0.032; n = 4 and 5, respectively, S1A Fig for representative examples). Higher levels H3K27ac and H3K4me3 and lower levels of H3K9me3 and H3k27me3 in knee compared to hip FLS were also observed (n = 4 and 5, respectively, S1B–S1E Fig). We then determined H3K27ac enrichment in knee and hip FLS by ChIP-qPCR and noted a trend toward higher in RA knee FLS relative to hip FLS (n = 6, S1F Fig). Of note, no other patterns of histone modifications distinguished the HOXD10 regulatory regions of RA knee and hip FLS. These data suggest that histone marks related to increased transcription in knee FLS could account for higher HOXD10 expression.

### H3K27acetylation dependent joint-specific HOXD10 transcript

**Fig 4:**
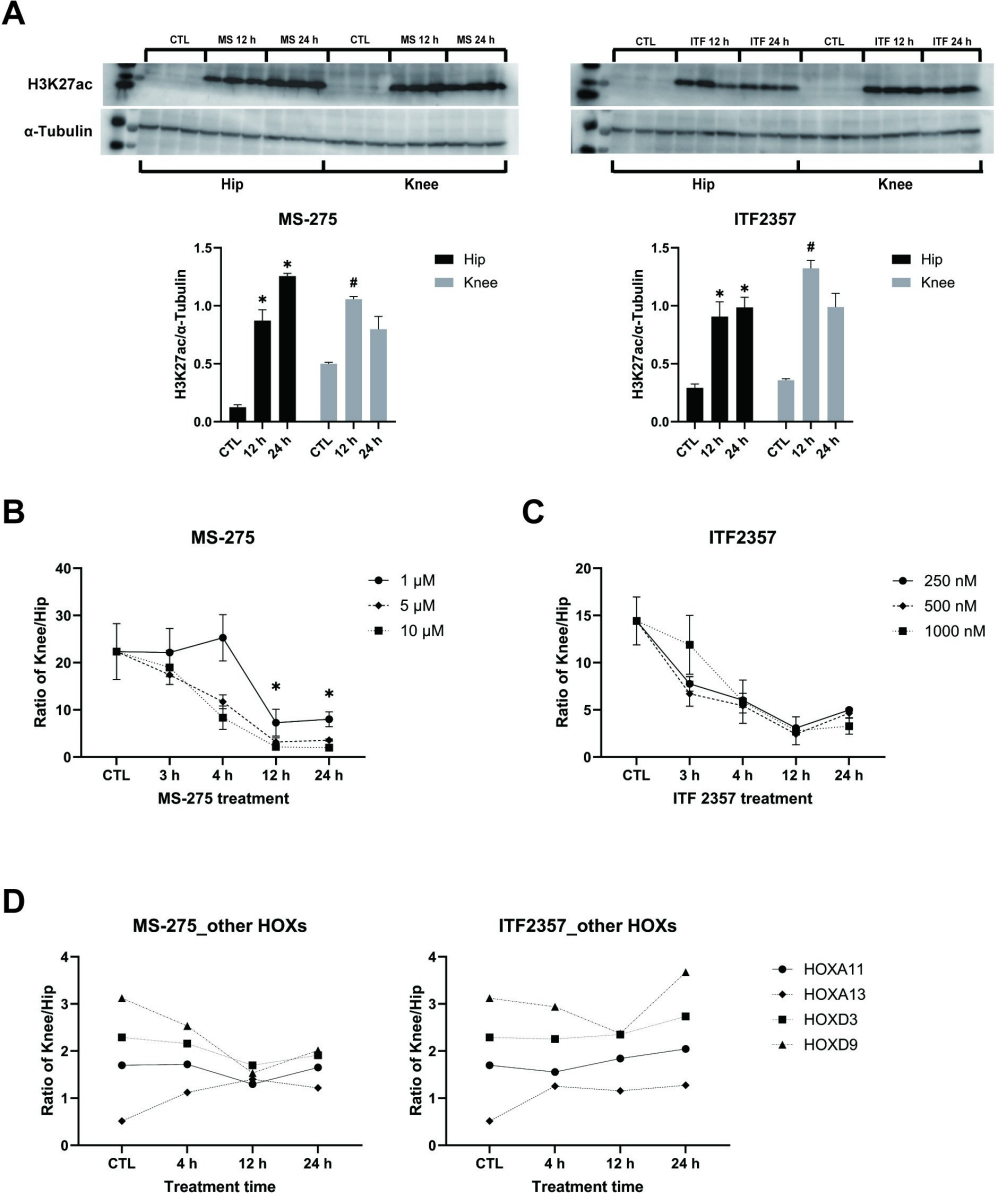
Contribution of H3K27ac in HOXD10 promoter to hip-knee differences. (A) RA FLS were treated with 10 μM MS-275 (HDAC1/3 inhibitor) (left panel) or 1000 nM ITF2357 (right panel) for 0–24 h (n = 3 separate lines for each experiment). Western blot analysis showed a significant increase of H3K27 acetylation after exposure to HDAC inhibitors. Data were presented as mean± SEM on bar graphs and analyzed using the two-tailed t-test. * and # P<0.05. Uncropped blots are included in S3 Fig. (B) After MS-275 treatment, HOXD10 mRNA level was measured at various time points by qRT-PCR (n = 3). The ratio of HOXD10 expression in knee FLS and hip FLS markedly reduced at 12h and 24 h. (C) After ITF 2357 treatment, HOXD10 mRNA level was measured at various time points by qRT-PCR (n = 3). The ratio of HOXD10 expression in knee FLS and hip FLS markedly reduced at 12h and 24 h. (D) Other HOX genes were measured after 10 μM MS-275 and 1000 nM ITF2357, respectively. The ratios for these HOX genes did not show significant differences after inhibitor treatment. (B-D) Data were analyzed by dividing gene expression of knee FLS by hip FLS, presented as mean± SEM and analyzed using the two-tailed t-test. *P<0.05.

To test whether hip-knee differences in HOXD10 were related to histone acetylation, we incubated hip and knee FLS with histone deacetylase (HDAC) inhibitors MS-275 (HDAC1/3) and ITF2357 (pan-HDAC). As expected, the HDAC inhibitors markedly increased H3K27ac levels (n = 3, Fig 4A). We then compared the knee-hip HOXD10 mRNA ratios after HDAC blockade. After 12 h, the ratio of HOXD10 expression in knee and hip FLS changed from 22-fold to 2.1-fold in 10 μM MS-275 and from 14-fold to 2.8-fold in 1000 nM ITF2357 (*P* = 0.0051 and 0.0127, respectively, n = 3, Fig 4B and 4C). In contrast, ratios for HOX genes that did not show knee-hip differences were not affected by the inhibitors (n = 3, Fig 4D). These findings indicate that H3K27ac plays a role in regulating the higher expression of HOXD10 in knee FLS.

### Effects of HDAC1 and HDAC3 inhibition and knockdown

**Fig 5:**
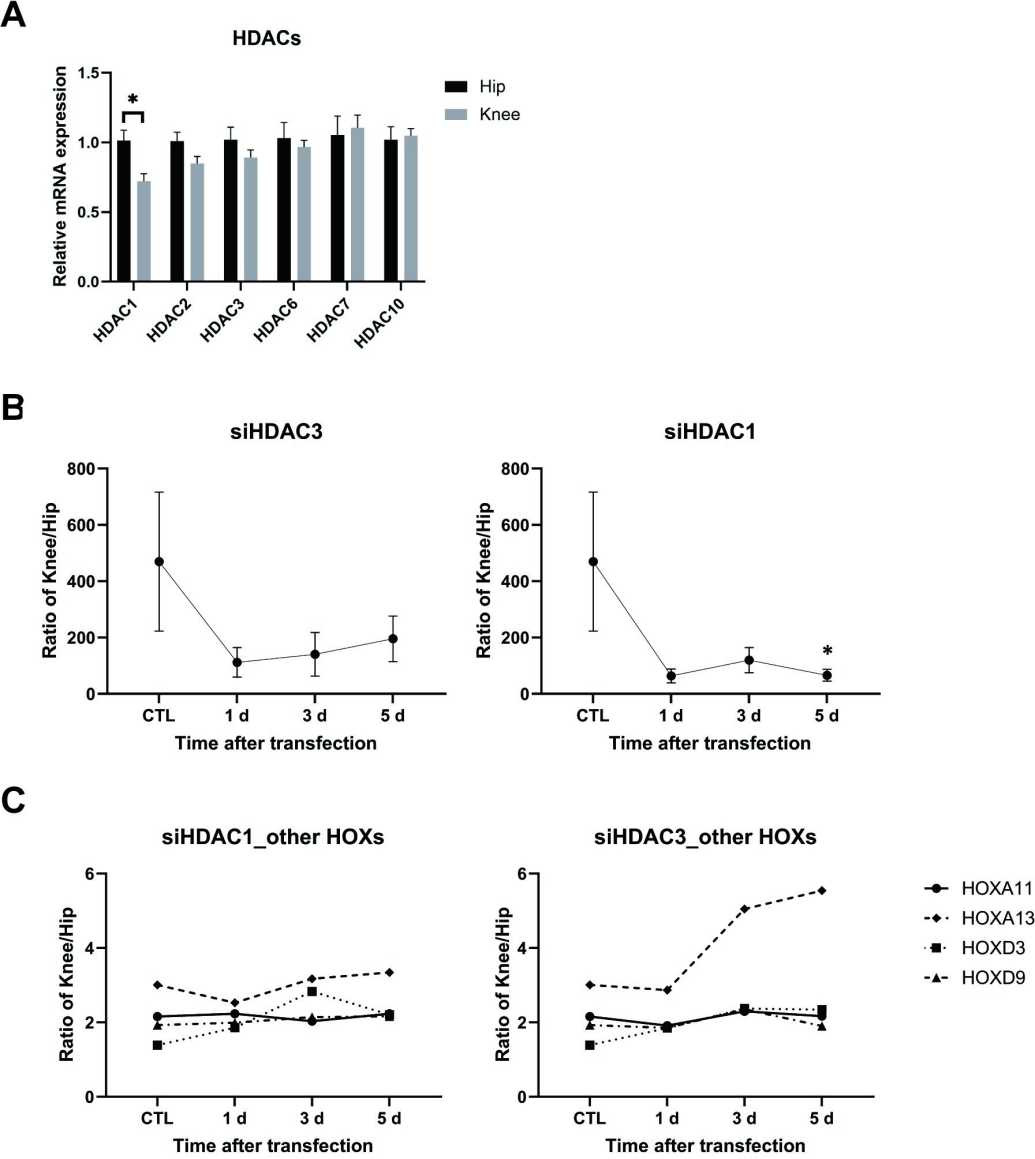
Effect of HDAC 1 and HDAC3 on HOXD10 expression. (A) Expression of HDAC RNA transcripts was measured by qRT-PCR in hip (n = 6) and knee (n = 6) FLS. HDAC1 expression was significantly higher in hip FLS compared with knee FLS. Data are presented as mean± SEM and analyzed using the two-tailed t-test. *P<0.05. (B) After transfection with siRNA targeting HDAC1, HDAC3 or non-targeting control (n = 3), HOXD10 mRNA was measured at various time points by qRT-PCR. The ratio of HOXD10 expression in knee FLS and hip FLS markedly reduced at 5 d after siHDAC1 transfection. (C) The ratios for the other HOX genes did not show significant differences after siRNA depletion of HDACs. (B, C) Data was analyzed by dividing gene expression of knee FLS by hip FLS, presented as mean± SEM and analyzed using the two-tailed t-test. *P<0.05.

Because MS-275 selectively inhibits HDAC1 and HDAC3, we profiled HDAC expression and function in FLS. We first analyzed expression of multiple HDAC levels by RT-qPCR and observed that only HDAC1 was significantly higher in RA hip FLS compared to knee FLS (n = 6, Fig 5A). We then determined which of the two HDACs is responsible for joint-specific HOXD10 expression using siRNA for HDAC1 or HDAC3. The HOXD10 ratio normalized in knee and hip FLS after 5 days, with a change from 469-fold to 65-fold for HDAC1 and 195-fold for HDAC3 (*P* = 0.0342 and 0.2500, respectively, n = 3, Fig 5B). HDAC 1 and HDAC 3 knockdown did not change the hip-knee ratio of other measured HOX mRNAs, except for a modest effect of HDAC3 deficiency on HOXA3 expression (n = 3, Fig 5C). Therefore, joint-specific HOXD10 expression patterns are related to lower HDAC1 function in knee FLS. HDAC1 depletion also increased expression of p21 and p16 mRNA in FLS derived from either joint (S4A and S4B Fig). More importantly, it also eliminated the significant difference and trend towards a difference between hip and knee.

## Discussion

We evaluated the mechanisms that account for markedly higher gene expression for HOXD10 in knee- compared to hip-derived FLS. Previous studies from our group as well as others demonstrated joint-specific epigenetic profiles including DNA methylation and transcriptome patterns [4, 5]. In our original paper on DNA methylation, two types of differential marks were noted that distinguished joint locations. First, some disease-independent epigenetic marks that distinguished hip and knee FLS were observed in RA as well as osteoarthritis (OA) FLS. Homeobox genes were particularly prominent and might contribute to embryonic development of individual joints and synovial homeostasis [25, 26]. Second, disease-specific marks were only observed between RA hip and knee joints, but not OA, and involved inflammatory and immunologically relevant genes. For example, IL-6 signaling via JAK-STAT and IL-17 signaling were differentially methylated in RA knee FLS compared to RA hip FLS and correlated with increased signaling in RA knee FLS [6].

In the present study, we extended these observations by focusing on RA and genes where the greatest differences between hip and knee were demonstrated. Our studies showed distinctive joint-specific gene expression patterns of HOX gene families in RA FLS and intact RA synovial tissue, including multiple HOXD and A genes. The HOX genes encode transcription factors expressed in specific body regions along the anterior to posterior (AP) body axis during embryonic development. For example, mutations in HOXD10 result in proximal shifts of the patella and alternation of the tibia and fibula [27, 28]. Location-specific HOX patterns affect joint development and might affect the characteristic patterns of joint involvement in synovitis.

Of the HOX genes that we profiled, HOXD10 was especially interesting because knee FLS expression was ~100–200 fold higher in hip FLS. Our focus on HOXD10 was also, in part, because HOXD10 functions are potentially important in the pathogenesis of RA. For example, this gene and other HOX genes regulate FLS migration, mitogen-activated protein kinase signaling, cell survival and cytokine expression [29, 30]. These differences could contribute to differences in disease severity in various joint location. Our goal was to determine how HOXD10 and other HOX genes are regulated by the inflammatory environment, the molecular mechanisms of differential expression of HOXD10 between hip- and knee-derived FLS. We discovered a unique epigenetic mechanism that is distinct from the recently described role of HOTAIR [30]. The differences in HOX gene expression, and most notably increased HOXD10 in knees, could be one contributing factor to the lower rates of arthroplasty in hips compared to knee [31].

Our initial studies to understand how these HOX genes and differential expression were regulated led us to explore the role of cytokines. The modulation of HOX gene expression by inflammatory cytokines has been observed in other cell types. For instance, IL-1β reduces expression of HOXA1, 3, 9, 10, and 11 in first trimester decidual cells [32]. In addition, several regulatory pathways, such as Wnt, fibroblast growth factor (FGF), and retinoic acid (RA) modulate HOX gene in other cells [33]. We did note that cytokines like TNF altered gene expression of multiple HOX genes in FLS, but the ratio of knee to hip HOX expression did not significantly change. Therefore, cytokine exposure is probably not the explanation for the observed joint differences.

Because cytokine exposure did not normalize HOXD10 expression between the two FLS types, we conducted studies assessing promoter activity and mRNA decay as well as the effect of factors such as, HOTAIR, histone methylation, and DNA methylation in hip and knee FLS. None of these could account for the prominent difference between the joints. Of interest, HOTAIR exhibits differential expression between upper and lower extremity synovial fibroblasts and represses HOXD and imprinted genes through modulation of H3K4me3 and H3K27me3 [5, 34]. Our result focused on only the lower extremities and showed only slightly higher HOTAIR levels in knee FLS compared to hip FLS. However, this did not explain the knee-hip differences because HOTAIR knockdown did not correct differential expression of HOXD10 transcript.

We then explored our epigenetic databases to identify the mechanism that might lead to the distinct HOXD10 depending on joint location. We discovered increased chromatin accessibility and higher H3K27ac and H3K4me3 histone marks along with and lower H3K9me3 and H3K27me3 peaks in the HOXD10 in the knee promoter versus hip promoter. The differential H3K27ac level was also consistent with ChIP-PCR studies using hip and knee FLS. Because of this, we focused our attention on H3K27ac to understand the mechanism underlying the joint-specific HOXD10 pattern.

To investigate whether H3K27ac in the HOXD10 promoter leads to different transcript levels between hip and knee FLS, we considered whether HDACs could be responsible for joint-specific gene expression. HDACs regulate the histone acetylation and gene transcription level together with histone acetyltransferase (HATs) during embryonic development [35]. HDACs also modulate several pathways involving cytokines, growth factors, and chemokines production relevant to [36, 37]. Further, HDACs are differentially expressed in some cells and tissues of RA patients [38].

We profiled HDACs in RA FLS and noted that HDAC1 was higher knee FLS. Using a small molecule inhibitor and siRNA, we confirmed that HDAC1 deficiency mitigated the knee-hip differences between hip and knee FLS. Our studies showed hip and knee HOXD10 levels are similar when HDACs, especially HDAC1, are blocked. In contrast, other HOX genes that lacked the differential marks or minimal differential gene expression did not change in response to HDAC inhibitors. Therefore, the marked differences between hip and knee FLS with regard to HOXD10 expression are related to differential histone marks and chromatin accessibility related to differences in HDAC expression and/or activity.

These studies have some limitations that need to be considered. First, FLS are cultured cells and might not reflect the situation in situ. The relevance of this observation was indicated by evaluating HOXD10 expression in intact synovium, where higher expression was also observed in the lining of knee than hip tissue. Although FLS have been in culture, they maintain their aggressive phenotype over many months and their distinctive epigenetic marks are stable [39–41]. We also have not profiled FLS obtained from other joints, and there could be alternative mechanisms such as differential HOTAIR expression for other joint locations like the upper extremity. Our data strongly suggest that the mechanisms of higher HOXD10 expression are related to epigenetic imprinting but we do not know whether cells are imprinted in the marrow and migrate to the correct joint or whether they are imprinted by the local environment after they take residence. Lastly, we only had limited information and are unable to stratify the findings based on clinical data. However, all patients had confirmed diagnosis of RA and had longstanding, severe disease requiring arthroplasty. Effects of concomitant therapy at the time of surgery on the epigenome has not been observed with cultured FLS, most likely because the cells have been in culture for multiple passages [42].

HOX genes can potentially participate in pathogenic processes related to RA. For example, HOXD10 regulates cell migration and Toll-like receptor, integrin, and p38/JNK signaling pathways [29]. It is possible that joint specific marks and gene expression such as the HOX genes contribute to inflammatory mechanisms. More intriguing, joint-specific HOX genes and imprinting might influence which joints are involved in arthritides like RA. By dissecting these mechanisms, we can potentially understand regional mechanisms of disease and its development as well as tailor therapy based on specific patterns of joint involvement.

## Supporting information

S1 Fig Epigenetic profile differences between hip and knee FLS. (A) Representative ATAC-seq peaks at the promoter of HOXD10 (chr2:176,980,555–176,981,754 (GRCh38/hg38)) in hip (n = 3, red) and knee (n = 3, blue) FLS. ATAC-seq analysis showed HOXD10 promoter in knee FLS was more accessible than hip FLS. (B-E) Representative H3k27ac (B), H3K4me3 (C), H3K9me3 (D), and H3K27me3 (E) ChIP-seq peaks at the promoter of HOXD10 in hip (n = 3, red) and knee (n = 3, blue) FLS. ChIP-seq analysis showed H3K27ac and H3K4me3 were enriched in knee FLS, and H3K9me3 and H3K27me3 were in hip FLS. (F) ChIP-qPCR analysis of H3K27ac enrichment at HOXD10 promoter region in hip (n = 6) and knee (n = 6) FLS. There was a trend towards higher levels of H3K27ac in knee FLS compared with hip FLS in HOXD10 promoter region. Data was normalized by input DNA, and IgG was used as control. Data was presented as mean± SEM and analyzed using the two-tailed t-test.
(TIF)

S2 Fig HOXD10 correlations with other genes. Data are based on the geTMM from reference 4 (gene length corrected trimmed mean of M-values). Pearson’s correlation analysis showed HOXD10 was positively correlated with MMP3 (A) and IL8 (D). n = 11; r, Pearson correlation coefficient; P, P-value.
(TIF)

S3 Fig Uncropped images from western blots. (A-B) Uncropped Western blot images are shown that correspond to Fig 4A.
(PDF)

S4 Fig The effect of HDAC 1 on cell cycle related genes. (A-B) After transfection with siRNA targeting HDAC1 or non-targeting control (n = 6 separate lines), p21 (A) and p16 (B) mRNA were measured at Day 5 by qRT-PCR. HDAC1 depletion induced both genes and eliminated the hip and knee FLS differences for p21 and p16 mRNA expression. Data are presented as mean± SEM and analyzed using the two-tailed t-test. *P<0.05.
(TIF)

S1 File Original data files. All original data from each figure were included in the Excel file.
(XLSX)
