# Supplementary figures and images for: Joint-specific regulation of homeobox D10 expression in rheumatoid arthritis fibroblast-like synoviocytes
Source: PLoS One. 2024 Jun 3;19(6):e0304530. doi: 10.1371/journal.pone.0304530 (PMC11146700; doi:10.1371/journal.pone.0304530)

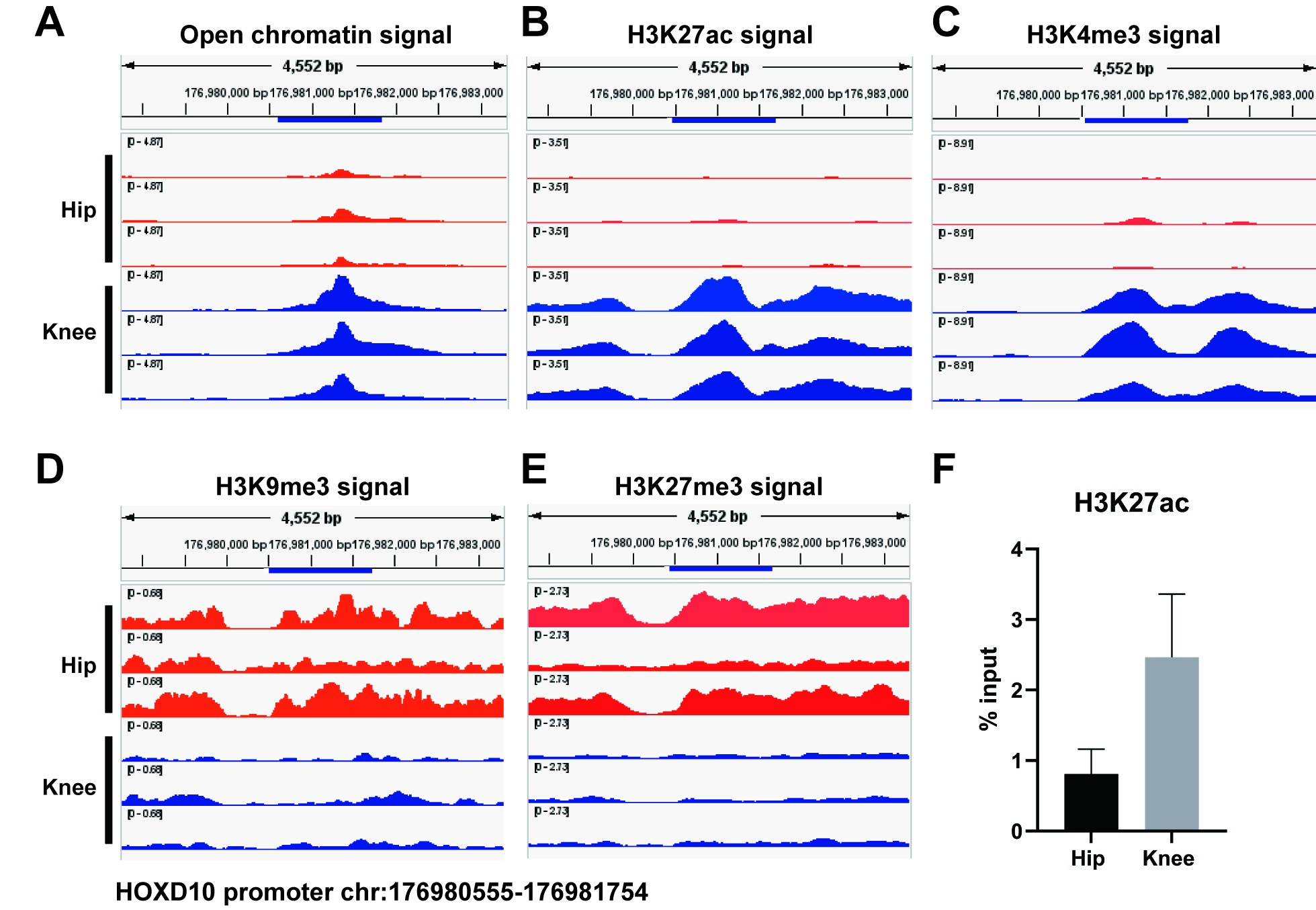

Supplement: S1 Fig — (A) Representative ATAC-seq peaks at the promoter of HOXD10 (chr2:176,980,555–176,981,754 (GRCh38/hg38)) in hip (n = 3, red) and knee (n = 3, blue) FLS. ATAC-seq analysis showed HOXD10 promoter in knee FLS was more accessible than hip FLS. (B-E) Representative H3k27ac (B), H3K4me3 (C), H3K9me3 (D), and H3K27me3 (E) ChIP-seq peaks at the promoter of HOXD10 in hip (n = 3, red) and knee (n = 3, blue) FLS. ChIP-seq analysis showed H3K27ac and H3K4me3 were enriched in knee FLS, and H3K9me3 and H3K27me3 were in hip FLS. (F) ChIP-qPCR analysis of H3K27ac enrichment at HOXD10 promoter region in hip (n = 6) and knee (n = 6) FLS. There was a trend towards higher levels of H3K27ac in knee FLS compared with hip FLS in HOXD10 promoter region. Data was normalized by input DNA, and IgG was used as control. Data was presented as mean± SEM and analyzed using the two-tailed t-test. (TIF) [file pone.0304530.s001.tif]

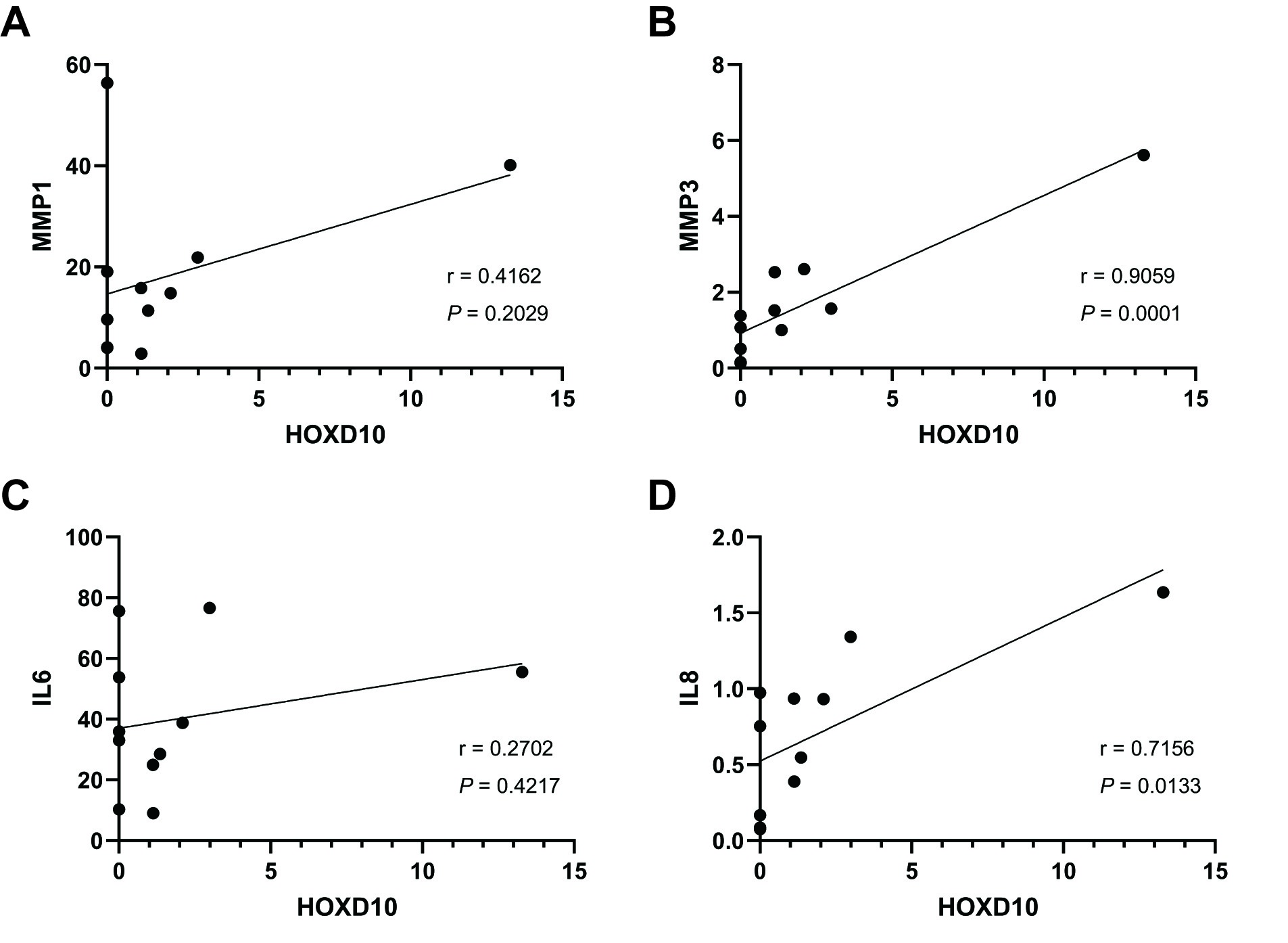

Supplement: S2 Fig — Data are based on the geTMM from reference 4 (gene length corrected trimmed mean of M-values). Pearson’s correlation analysis showed HOXD10 was positively correlated with MMP3 (A) and IL8 (D). n = 11; r, Pearson correlation coefficient; P, P-value. (TIF) [file pone.0304530.s002.tif]

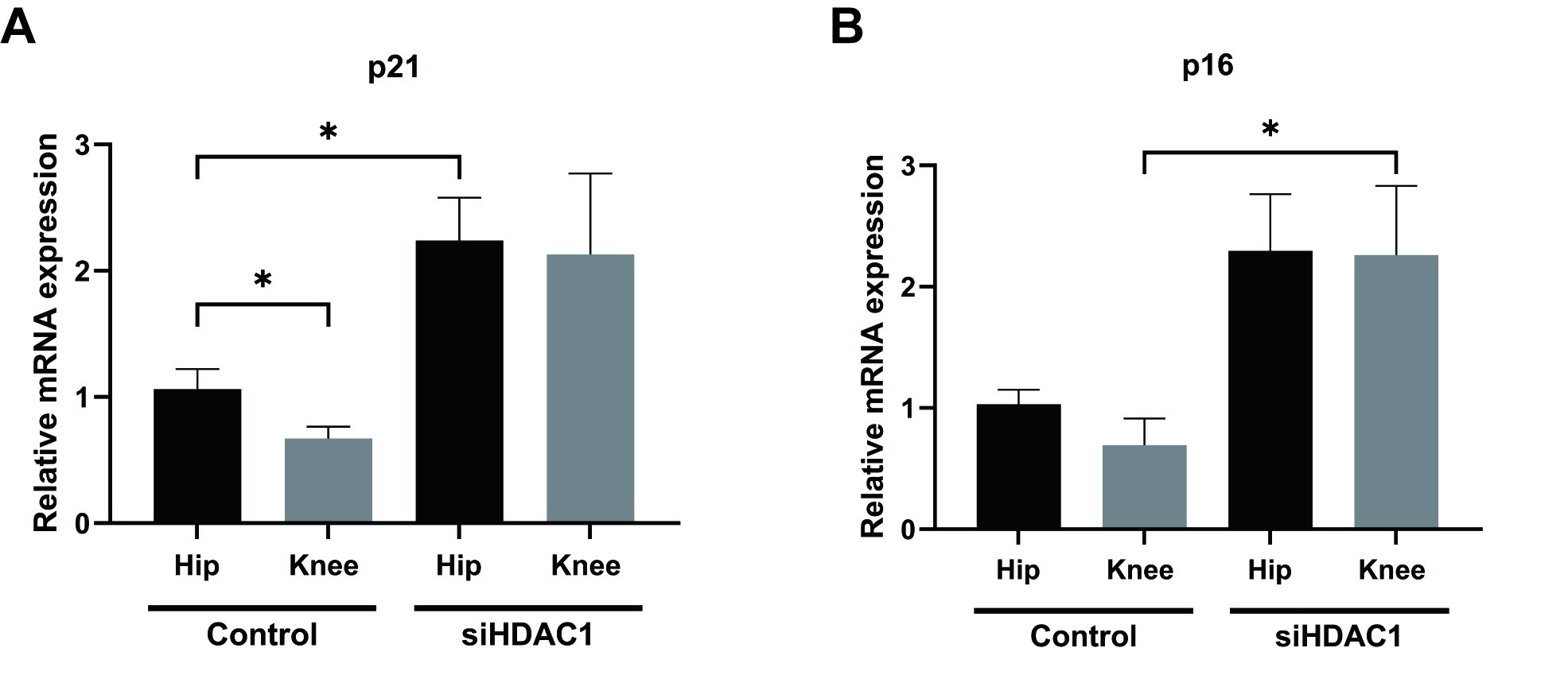

Supplement: S4 Fig — (A-B) After transfection with siRNA targeting HDAC1 or non-targeting control (n = 6 separate lines), p21 (A) and p16 (B) mRNA were measured at Day 5 by qRT-PCR. HDAC1 depletion induced both genes and eliminated the hip and knee FLS differences for p21 and p16 mRNA expression. Data are presented as mean± SEM and analyzed using the two-tailed t-test. *P<0.05. (TIF) [file pone.0304530.s004.tif]
