# Supplementary material for: Joint-specific regulation of homeobox D10 expression in rheumatoid arthritis fibroblast-like synoviocytes
Source: PLoS One. 2024 Jun 3;19(6):e0304530. doi: 10.1371/journal.pone.0304530 (PMC11146700; doi:10.1371/journal.pone.0304530)
Supplement: S3 Fig — (A-B) Uncropped Western blot images are shown that correspond to Fig 4A. (PDF) [file pone.0304530.s003.pdf]

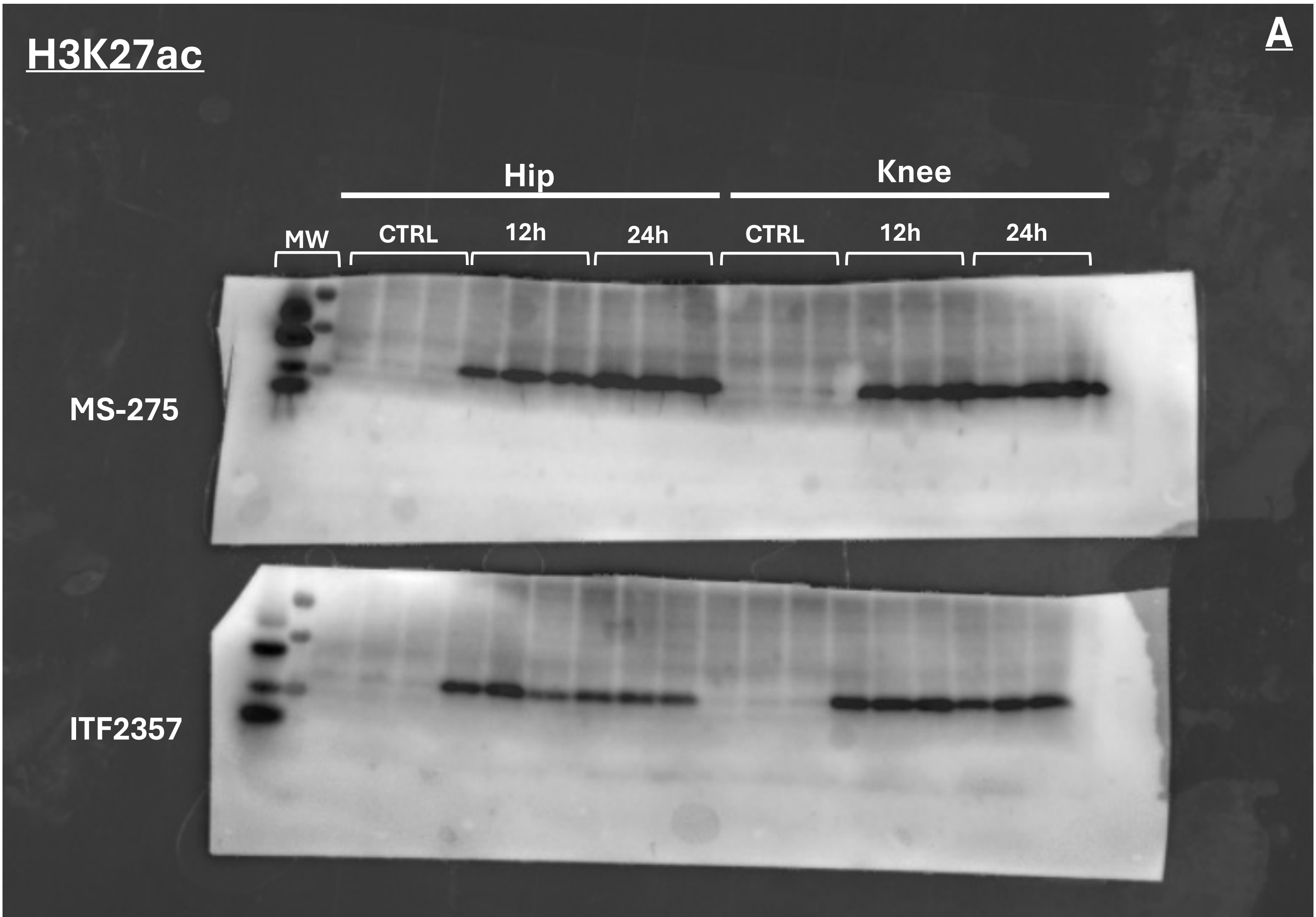

These Western blot raw image correspond to Figure 4A (top left and top right)
The images were processed by G:BOX Syngene equip

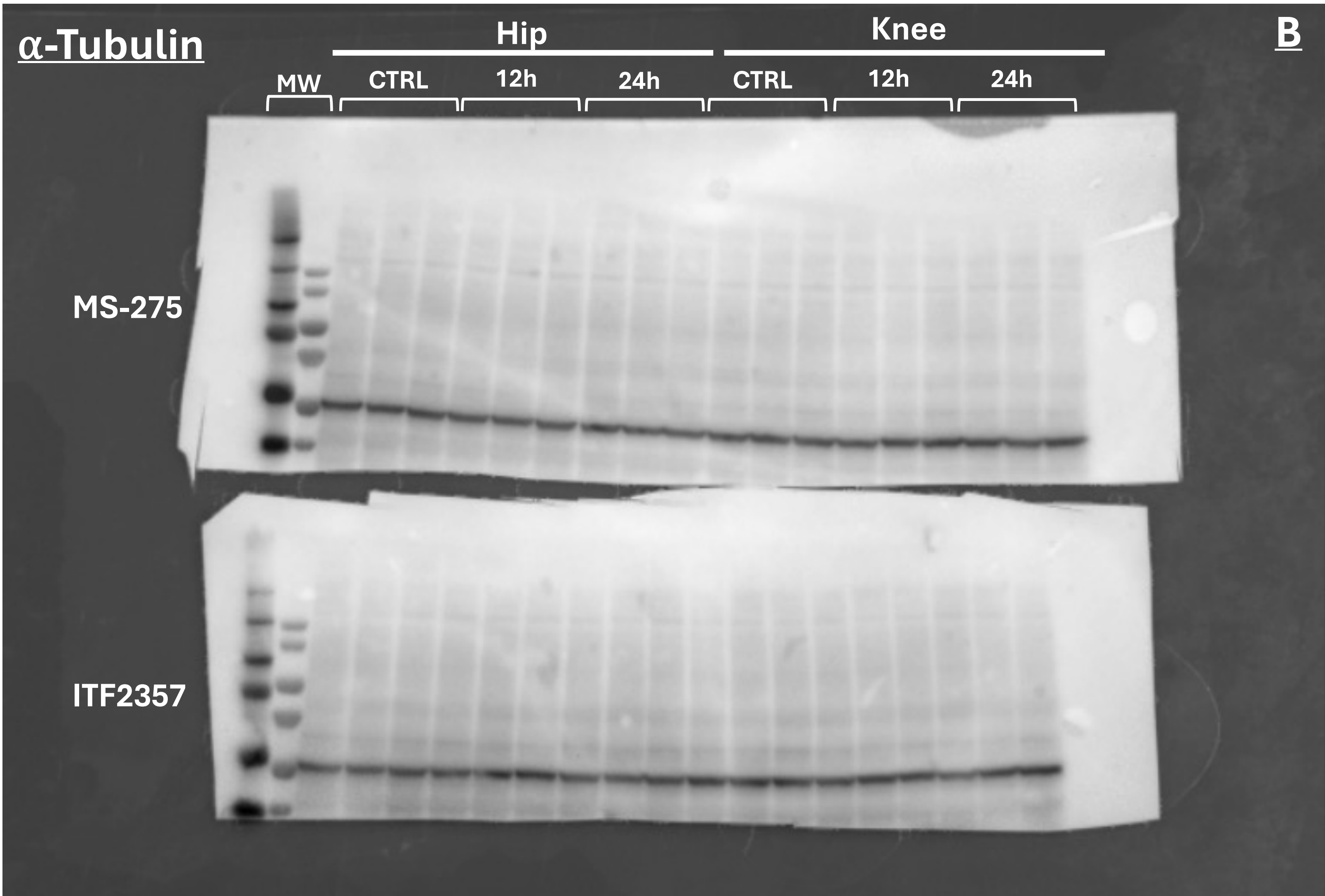

These Western blot raw image correspond to Figure 4A (bottom left and bottom right)
The images were processed by G:BOX Syngene equip
